# A mixed methods study on patients’ and healthcare professionals’ understanding of the graphical presentation of patient-reported outcome data at an inpatient unit for psychosomatic medicine

**DOI:** 10.1186/s41687-026-01061-w

**Published:** 2026-04-11

**Authors:** Anna Margarete Maria Thurner, Jonas Egeter, Franziska Gross, Fanny L. C. Loth, Timo Schurr, Bernhard Holzner, Barbara Sperner-Unterweger, Katharina Hüfner, Johannes Maria Giesinger

**Affiliations:** 1 University Hospital of Psychiatry II, Department of Psychiatry, Psychotherapy, Psychosomatics and Medical Psychology, Medical University of Innsbruck, Innsbruck, Austria; 2 Professorship for Psychological Diagnostics and Intervention, Catholic University of Eichstätt-Ingolstadt, Eichstätt, Germany; 3 University Hospital of Psychiatry I, Department of Psychiatry, Psychotherapy, Psychosomatics and Medical Psychology, Medical University of Innsbruck, Innsbruck, Austria

**Keywords:** Patient-reported outcomes, Graphical presentation, Psychosomatic patients, Health care professionals, ePROM

## Abstract

**Background:**

Patient involvement is a key goal in modern healthcare, and quality of care improves with effective information flow between patients and healthcare professionals (HCPs). Patient-reported outcomes (PROs) are essential in patient-centered care. Electronic PRO monitoring (ePROM) involves collecting, analyzing, and documenting patient self-ratings, enabling treatment monitoring, screening, quality assurance, and research. The aim of this study was to investigate patients’ and HCPs’ understanding of the graphical presentation of longitudinal ePROM bar charts and the patients’ perspective on various modes of access to such data (e.g. paper reports, electronic formats, or HCP consultations).

**Methods:**

Semi-structured interviews with 40 patients and 13 HCPs involved presenting longitudinal ePROM bar charts. Participants answered questions about changes and current states of four constructs (disease understanding, treatment effectiveness, depression, anxiety). They also interpreted the graphs and answered questions regarding report preferences, pros and cons of different report types, preferred assessment frequency, reactions to problems (e.g., red graph), and support requests for problematic results.

**Results:**

Results showed that 80% of patients correctly interpreted the graphs (9/12 questions), with only one patient answering fewer than 50% correctly. 84.7% of HCPs answered at least 15/16 questions correctly. A significant association was found between patients’ understanding of ePROM results and their educational level. Most patients preferred receiving their ePROM reports through personal meetings with a written report for memory support.

**Conclusion:**

Both patients in psychosomatic medicine wards and HCPs demonstrated a high level of understanding of ePROM results, with patients' educational levels influencing accuracy. Patients strongly preferred receiving ePROM reports through personal contact, suggesting that future studies should explore the effects of this report method.

**Supplementary Information:**

The online version contains supplementary material available at 10.1186/s41687-026-01061-w.

## Background

A key component of patient-centered healthcare is the use of patient-reported outcomes (PROs) measures to evaluate the impact of health conditions and their treatment from the patient’s perspective. While the term PRO has been introduced only in the last two decades [1, 2], self-report questionnaires have been used in patients with psychological disorders for over a century in both clinical practice and clinical research [1]. The long history of PRO use illustrates persistent challenges, such as ensuring patients understand the information, integrating their responses meaningfully into care, and presenting results in clinically actionable ways, which remain highly relevant for today’s implementation of electronic patient-reported outcome monitoring (ePROMs). With the advent of widely available IT infrastructure, PRO assessments have increasingly moved away from traditional paper-pencil assessments to electronic modes of assessment (e.g., via tablet PCs or mobile phones [2], which proves especially beneficial in daily practice where immediate availability of PRO results is crucial [3, 4].

In clinical psychology and psychiatry, ePROM has been introduced for longitudinal assessment in daily practice to monitor, patients’ mental health status and related health aspects [5, 6]. While such data collected via ePROM is still frequently only accessible to health professionals, efforts across medical fields have been made to make ePROM results accessible also to patients to foster patient involvement and shared decision-making [7]. However, this also introduces challenges on how ePROM results should be made accessible for patients. Issues such as data privacy concerns and differing patient preferences complicate how providers share ePROM data [4]. While paper reports remain familiar and tangible for many patients, especially those less digitally literate, electronic formats offer advantages in accessability and ease of integration into modern health systems [8]. These highlights the need to understand patients and healh care professionals (HCPSs) perspectives when designing how ePROM data should be shared. Another key aspect of the accessibility of ePROM data is facilitating their understanding and interpretation. Because modern ePROM systems increasingly aim to support real-time therapeutic decision-making, both patients and clinicians must be able to interpret the displayed information accurately. Thus, the way ePROM data are graphically presented becomes a central component of accessibility, ensuring that patients, regardless of psychological symptom burden, can engage with their own health information [9]. Clear and intuitive visualizations are therefore essential to enable shared decision-making and strengthen patient involvement [10] in routine psychiatric care. Therefore, several studies investigated how graphical formats presenting ePROM data are perceived and understood by stakeholders such as patients or HCPs. Such studies investigated data presentation in two basic settings: group-level data to communicate study results [9–12] or patient-level data in a clinical practice setting [11–14]. Most of such research has been done in patients with somatic illnesses, leaving the question of specific challenges in patients with psychological disorders unanswered. However, to date, there is a notable lack of comparable investigations involving psychosomatic patients. Findings may derive from somatic illness and not be directly transferable to psychosomatic populations. The present study aims to address this gap by examining the phenomenon within a psychiatric framework, thereby contributing to a more differentiated understanding of its relevance and manifestation across clinical contexts. For future clinical procedures in which patients routinely receive their PRO results, it is important to ensure that these data can support self-evaluation and aftercare. If patients receive information about their health status, it must be carefully considered whether they can truly understand the results when shared. This is particularly relevant for psychiatric patients, as their symptoms are often experienced in a different context than patients with somatic illnesses and cognitive impairments assosciated with mental disorders may affect comprehension [15]. To the best of our knowledge, only a single study [11] investigated the optimal presentation of patient-level PRO data from routine PRO monitoring in patients with psychological disorders. Based on their findings, Kristensen et al. emphasized the need for a clear and unambiguous graphical presentation of ePROM data and suggested the use of bar or line charts, including elements that aid interpretation for the use in clinical consultations. Moreover, their work demonstrated that involving patients directly in the data collection methods, and graphical display formats can generate concrete, unambiguous, and easily understandable presentations that align with patient preferences and support clinical implementation [16]. Kuijpers et al. investigated which graphical presentation formats patients and healthcare professionals could interpret most accurately. In their study, various formats were tested. The results indicated no significant difference of objective interpretation accuracy between these formats, in cancer patients [12]. It is important to note that the preferred graphical presentation style chosen by patients may not necessarily align with the presentation style they can interpret the most accurately [17].

To address this critical gap in the field, our study systematically evaluated how psychosomatic inpatients and healthcare professionals understand the graphical presentations of longitudinal ePROM data, and explored patient preferences for data access. In line with the findings of Kristensen et al. we relied on bar charts for graphical presentation [11, 16, 18, 19] to support accurate presentation of results. Our study pursued three main objectives:

1. To evaluate patients’ and HCPs’ understanding the graphical presentation of longitudinal ePROM data.
2. To assess patients’ preferences regarding different modes of ePROM assessment and feedback reports and the frequency of willingness to fill in the questionnaire.
3. To explore patients’ qualitative feedback on the perceived advantages and disadvantages of the various report formats (e.g. paper reports, electronic formats, or HCP consultations).

## Methods

### Sample and setting

For our cross-sectional, mixed-method study, we recruited patients and HCPs at the Department of Psychiatry, Psychotherapy, Psychosomatics and Medical Psychology, Medical University of Innsbruck (Austria). Eligible were all inpatients who had participated previously in the routine ePROM assessments at the department and had completed at least four weekly assessments during their inpatient stay. Further inclusion criteria were a minimum age of 18 years, no cognitive impairments compromising questionnaire completion, sufficient command of German, and written informed consent. The clinical team confirmed participants had no cognitive impairments that would prevent ePROM completion or interviews. For the HCP sample, any staff member involved in patient treatment was eligible for participation. The routine ePROM assessments at the department were introduced in 2014 and details on the implementation can be found elsewhere [20]. Routine data collection included sociodemographic and clinical patient characteristics and ePROM measures assessing a range of symptoms and functional health. EPROM relied on the software CHES for data collection and graphical presentation of ePROM results [20, 21]. To capture meaningful longitudinal data, four weekly assessments were conducted, allowing fluctuations in symptoms and patient-reported outcomes to be observed over time. The study was underpinned by a qualitative content analysis approach and data saturation was addressed as part of the study design. Participants did not review or provide feedback on their interview transcripts.

### Study procedure and assessment

Two female trained research assistants, a graduate student and a PhD student in psychology, used a purposive sampling approach to identify eligible patients for face-to-face study participation and approached healthcare professionals (HCPs) at the department. The recruitment and data collection took place over 17 months, and the bar charts display ePROM assessments covering a period of 4 weeks.

The participants were informed that the researchers were conducting the study as part of an effort to improve PRO feedback for patients. The patient assessment included a quantitative and qualitative part. The quantitative part focused on the accuracy of the interpretation of the graphical presentations of the ePROM data. Additionally, we investigated the frequency with which patients would be willing to complete the questionnaires and the preferred report method for their ePROM results. In the qualitative part, we used semi-structured interviews, without visual or audio recording, to assess the patients’ perspective on (dis)advantages of the various report methods (“Can you think of any advantages or disadvantages of the individual ePROM report variants?”), on highlighted problems (“What do you think a problem (red bar) would trigger in you?”) and required support (“What support would you like to receive?”). Field notes were taken during the interviews, which lasted approximately 30 min each. Audio recording was not used due to the challenges associated with psychiatric settings, where recording can increase discomfort and non-participation rates. Participant feedback on the study findings was not obtained.

#### Graphical presentation style

For our study, we selected four domains from the measures included in the ePROM to be displayed in the graphical presentation format. These four scales were chosen from three questionnaires, representing two central and complementary concepts: two scales assessing core psychological outcomes (depression and anxiety) and two scales capturing patients’ understanding of their disease and illness perception. The following questionnaires were included in this study:

- “Understanding of disease” and “Help through treatment” from the Brief Illness Perception Questionnaire (B-IPQ) [22]. These two single items form separate scales and are answered on a 10-point rating scale. High scores indicate a good understanding of the disease or a high expectation that the treatment can improve the patients’ condition.
- The “Depression” score calculated from Beck’s Depression Inventory (BDI-II) indicates the severity of depressive symptoms on a scale ranging from 0 to 63, with high scores indicating high levels of depression.
- The “Anxiety” score was obtained from the State-Trait-Anxiety Inventory (STAI), from which we selected the state anxiety scale that indicates current levels of anxiety on a scale from 20 to 80. High scores indicate high levels of state anxiety.

**Fig. 1:**
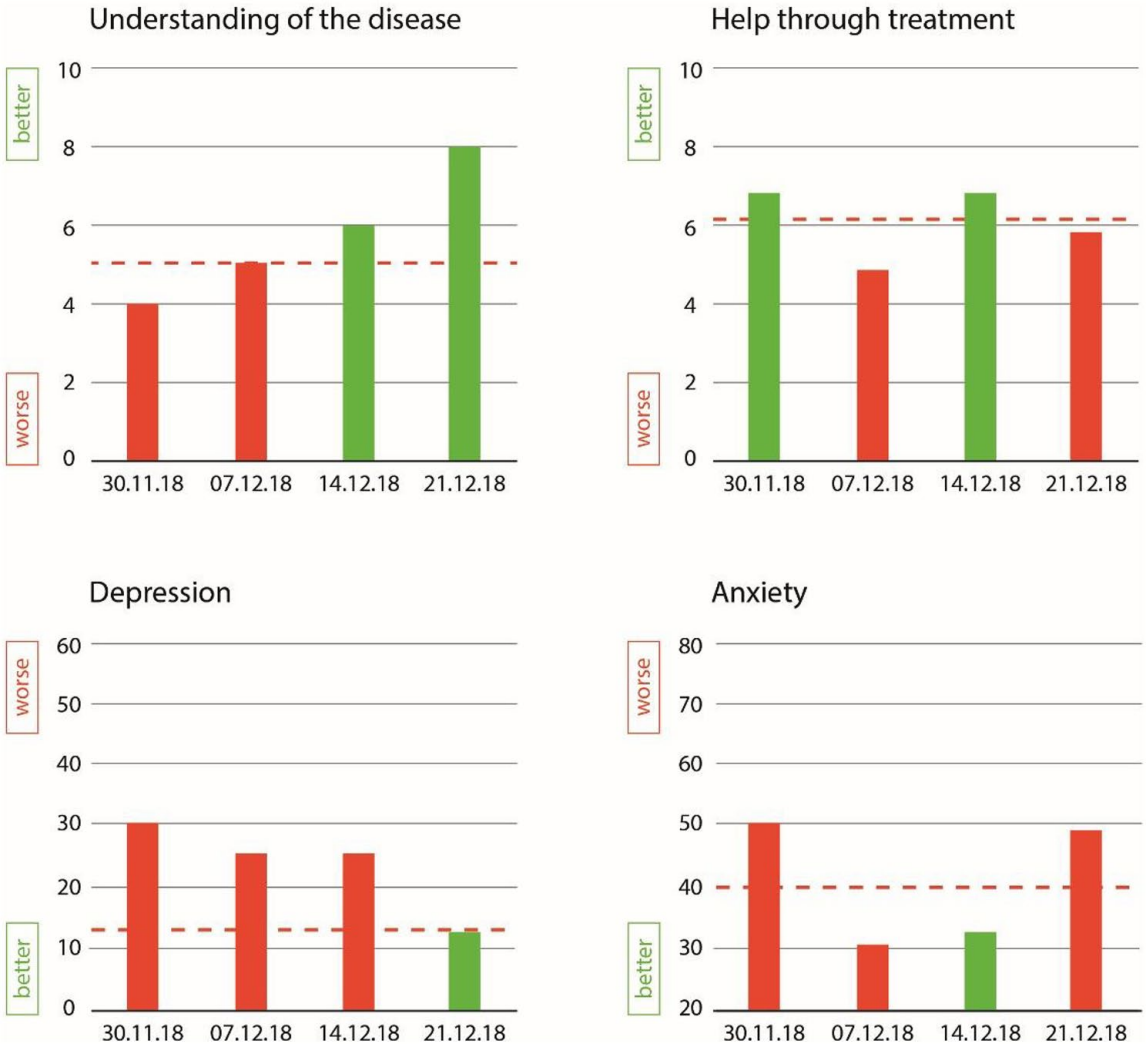
Graphical presentation of ePROM results as shown to the patients and HCPs

For each of these scales, we showed four assessment time points as bar charts with time points on the x-axis and the ePROM score points on the y-axis. Clinically meaningful scores were highlighted in red, while others were green. Patients were shown their results from the previous four assessments they had completed, while HCPs were all shown the same scores and several time points from a fictitious patient. Figure 1 shows an example of the presentation format. The decision to use bar charts for the graphical presentation of ePROM data was based on prior evidence from the literature [11, 12, 16, 18, 19].

#### Evaluation of interpretation accuracy

Participants were presented the ePROM results and asked the following questions for each of the four scales to assess interpretation accuracy:

- Has the ePROM score improved, worsened, or remained stable **between the first and the last time point** for the following scale?
- Has the ePROM score improved, worsened, or remained stable **between the most recent time points** for the following scale?
- Please indicate in which areas there is a problem on **the most recent date** according to the chart.
- HCP participants were also asked: Has the ePROM score improved, worsened, or remained stable **across all time points** for the following scale?

The research assistant coded participant responses as correct or incorrect. Patients could achieve a maximum of 12 correct answers, while HCPs could achieve a maximum of 16 correct answers.

#### Patient preference for ePROM assessment and report

Furthermore, patients were asked questions about their willingness to complete questionnaires during the inpatient stay (response options: never, every week, every two weeks, once a month, less than once a month) and at home (response options: never, once a month, every three weeks, every six months, once a year). The preferred patient report method was investigated quantitatively and qualitatively. For each evaluated report method (paper report, electronic report via e-mail, conversation with HCP, not receiving any results), we asked patients for their preference (yes/no). Also, we collected qualitative information on the (dis)advantages of each method and asked: “What do you think a problem (red bar) would trigger in you?” separately for two settings, at home and while in the hospital.

### Data analysis

Socio-demographic and clinical patient characteristics are reported as relative and absolute frequencies, as well as means and standard deviations. Interpretation accuracy, i.e. the number of correct responses, was calculated for each participant and is reported descriptively. The impact of patients’ educational level on the number of correct responses was analyzed with a Mann-Whitney test comparing patients with lower education (compulsory school or vocational education) against those with higher education (a-levels or university degrees). Qualitative interview data were categorized independently by two reviewers who developed their independent category systems, harmonized it in a consensus discussion, and then recoded the data. For each of the categories, we provide the frequency along with examples of patient comments. Themes were derived from the data using an inductive approach. Microsoft Excel was used to organize and manage the data. The qualitative data were analyzed using thematic analysis. Coding was conducted in an open and iterative manner with regular team discussions to ensure consensus, and data saturation was reached when no new themes emerged from the interviews. First, all interview transcripts (or written responses) were read to gain familiarity with the data. In an open coding process, each relevant segment of text was labeled with a code reflecting its meaning. This initial coding allowed identification of significant ideas and patterns directly emerging from the responses. Next, codes were discussed among the research team in regular meetings to refine them and resolve discrepancies. Related codes were then grouped into broader categories, and sub-codes were organized under overarching themes in an iterative process, repeatedly returning to the data to ensure accuracy and completeness. Finally, the resulting themes were clearly defined, named, and supported with illustrative participant quotations to convey the main patterns and meanings in the dataset. This approach ensures that the qualitative analysis is systematic, transparent, and grounded in the data. All statistical analyses were done with the software SPSS 21.0.0.0 [23].

## Results

### Participant characteristics

For our study, we recruited 40 patients (85.0% women) with a mean age of 34.8 years (range 18–66) at the ward for psychosomatic medicine of the University Hospital, Psychiatry II at the Medical University of Innsbruck. The two most frequent diagnoses were eating disorders (37.5%) and somatoform disorders (32.5%). Patient assessments were done at the hospital, except for two patients who were interviewed via telephone after discharge because a face-to-face interview was impossible due to administrative reasons. Detailed patient characteristics are shown in Table 1.

**Table 1:** Patients characteristics (*N* = 40)

		Mean (SD; Range)
Age		34.75 (13.9; 18–66)
Gender		N (%)
	Female	34 (85.0)
	Male	6 (15.0)
Marital Status		
	Single	29 (72.5)
	Married	3 (7.5)
	Partnership	3 (7.5)
	Divorced/separated	5 (12.5)
Educational Status		
	Compulsory or vocational education	15 (37.5)
	A-levels	16 (40.0)
	University degree	9 (22.5)
Employment Status		
	Full-time employment	10 (25.0)
	Part-time employment	6 (15.0)
	Unemployed	16 (40.0)
	Retired	2 (5.0)
	In training	6 (15.0)
Main diagnosis according to ICD-10 (WHO, 2016)		
F10-F19	Mental and behavioral disorders due to psychoactive substance use	2 (5.0)
F30-F39	Mood (affective) disorders	8 (29.0)
F40-F48	Neurotic, stress-related and somatoform disorders	13 (32.5)
F50-F59	Behavioral syndromes associated with physiological disturbances and physical factors	15 (37.5)
F60-F69	Disorders of adult personality and behavior	2 (5.0)

Among the 13 participating HCPs, the mean age was 40.6 years (range 22–59), and 11 (84.6%) were women. On average, the professional experience was 10.4 years (range 0.2–35). The professional background was nursing (7 participants, 53.8%), psychology/psychiatry/psychotherapy/medicine (4 participants, 23.1%), or social work, physiotherapy (1 participant each, 7.7%).

### Accuracy of interpretation

Most patients (80.0%) interpreted at least 9 out of 12 questions correctly, and only one answered less than 50% correctly. Similarly, most HCPs (84.7%) answered at least 15 of 16 questions correctly. Detailed results are shown in Table 2. There was a statistically significant difference between patients with higher education (a-levels or above) and patients with lower education (less than a-levels), with higher educated patients having on average 0.7 questions more correct (*p* = 0.024).

**Table 2:** Results on interpretation accuracy of patients and HCPs

Patients		Health Care Professionals	
Correct answers	*N(%)*	Correct answers	*N(%)*
12/12	10 (25.0)	16/16	6 (46.2)
11/12	11 (27.5)	15/16	5 (38.5)
10/12	4 (10.0)	13/16	1 (7.7)
9/12	7 (17.5)	11/16	1 (7.7)
8/12	3 (7.5)		
7/12	1 (2.5)		
6/12	3 (7.5)		
3/12	1 (2.5)		

### Preferences for report formats

Regarding the frequency of questionnaire completion, most patients (85.0% reported to prefer weekly assessments while in the hospital, and 77.0% of patients preferred monthly assessments at home. See details in Table 3. While in the hospital, most patients preferred to get their report via a personal meeting with the HCP with an additional report (55.0%), followed by report in the form of a paper report (20.0%). At home, patients preferred report in the form of an electronic report (31.6%) and a personal meeting with a report (28.9%). For further details, see Table 4.

**Table 3:** Frequency of willingness to fill in the questionnaire

Questionnaire frequency	At ward (*N* = 40)	At home (*N* = 39)
	*N* (%)	*N *(%)
Once a week	34 (85.0)	0 (0.0)
Every two weeks	5 (12.5)	0 (0.0)
Once a month	0 (0.0)	30 (77.0)
Less than once a month	1 (2.5)	0 (0.0)
Every three months	0 (0.0)	3 (7.7)
Every six months	0 (0.0)	1 (2.6)
Once a year	0 (0.0)	2 (5.1)
Never	0 (0.0)	3 (7.7)

**Table 4:** Prefered report method (quantitative and qualitative results)

Prefered report methode - quantitative analysis			Report methode (dis)advantages (three most frequent mentiones categories) – qualitative analysis	
	At hospital (*N* = 38)	At home (*N* = 38)	Advantages	Disadvantages
	*N* (%)	*N* (%)		
Paper Report	8/38 (20.0)	7/38 (18.4)	Availability, clarity, not relying on IT for data access	Risk of losing the paper report, lack of personal contact with HCP, poor economic/ecological efficiency
Electronic Report	1/38 (2.5)	12/38 (31.6)	Quick access, good availability, economic/ecological efficiency	IT resources and capabilities, no personal contact with HCP, concerns about data protection
During ward round	5/38 (12.5)	n/a	Personal contact, information exchange between HCPs, good comprehensibility	Lack of privacy, requiring too much time, need to be present personally
Personal meeting with report	22/38 (55.0)	11/38 (28.9)	/	/
Personal meeting without report	0/38 (0.0)	0/38 (0.0)	Cosultation itself, good comprehensibility of ePROM data, privacy	Time required, confrontation with the condition, in-person presence required
No report	2/38 (5.0)	8/38 (21.1)	Not beeing confronted with one’s condition, no confrontation with HCPs, no unnecessary information	Lack of access to information, lack of motivation to complete the questionnaire, emotional distress caused by not knowing the results

#### Qualitative feedback

In the semi-structured interviews, the 40 patients provided a total of 596 comments on the possible (dis)advantages of the different report methods for the ePROM results and related topics. More details on quantitative and qualitative results for patients preference, see Table 4.

##### Report in the form of a paper report

About half of the patients (*n* = 23/58.9%) mentioned the advantage of the paper report’s availability (e.g., “I have something in hand, and it is good memory support”), followed by the clarity such reports provide (*n* = 6/13.4%) (e.g., “This would be as simple as possible and common for anyone”) and the convenience of not relying on IT for the data access (*n* = 5/12.8%) while obtaining important information (*n* = 3/7.7%). Comments on the disadvantages were the risk of losing the paper reports (*n* = 10/25.6%), the lack of personal contact with the HCP (*n* = 6/15.4%), less efficiency in terms of economic/ecological resources (*n* = 6/15.4%) (e.g. waste of paper, needing more time) and poor archivability (*n* = 5/12.8%) (e.g., “Too many notes lying around”). Other issues included problems with delivery of reports (*n* = 3/7.7%), poor data protection (*n* = 2/5.1%) and being overburdened by the information (*n* = 1/2.6%).

##### Report in the form of an electronic report

Quick access (n = 12/33.3%) (e.g., “I do get the results faster”), good availability (n = 7/19.4%) (e.g.,”I have it with me on my phone”), efficiency in terms of economic/ecological resources (*n* = 4/11.1%) (e.g. “It is not a waste of paper”), good archivability (*n* = 4/11.1%) (e.g., “It saves space”), and subjective preference (*n* = 4/11.1%) were named as advantages of electronic delivery of findings. Other factors mentioned were ease of transfer to third parties (*n* = 2/5.6%), adaptability of graphical display (*n* = 1/2.8%), and being a contemporary approach (*n* = 1/2.8%). Disadvantages mentioned included the need for IT resources and capabilities (*n* = 12/33.3%) (e.g., “I am clumsy with the computer.”), no personal contact with HCP (*n* = 8/22.2%), concerns about data protection (*n* = 5/13.9%) (e.g., “I do not know who has access to the data”), and loss of the report (*n* = 5/13.9%) (e.g., “Delivery might not work”). Poorer comprehensibility (*n* = 2/5.6%), subjective preference (*n* = 2/5.6%), and the need to actively search for information (*n* = 1/2.8%) were also mentioned.

##### Report during the ward round

The benefits of providing ePROM monitoring report at the ward round were mentioned as personal contact (n = 15/39.5%) (e.g., “I can discuss my problems and get advice or medication”), information exchange between HCPs (n = 8/21.1%) (e.g.” I get are report from different points of view.”) and good comprehensibility (*n* = 6/15.8%). In addition, patients mentioned the information gain for HCPs (*n* = 5/13.2%) and the information gain for patients (*n* = 3/7.9%), the available support (*n* = 1/2.6%), the reflection on their condition (*n* = 1/2.6%). Disadvantages mentioned by patients were lack of privacy (*n* = 17/44.7%) (e.g., “Everyone in the ward round can listen”), requiring too much time (*n* = 4/10.5%), the need to be present personally (*n* = 3/7.9%), time pressure during the ward round (*n* = 2/5.3%), and a too long waiting time for access (*n* = 1/2.6%).

##### Report in the form of a personal meeting

The most frequently cited advantages of ePROM monitoring report in the form of personal meetings with the HCP were the consultation itself (n = 19/52.8%) (e.g., “Problems can be addressed immediately”), good comprehensibility of ePROM data (n = 14/38.9%), and privacy (n = 12/33.3%) (e.g. “direct consultation is possible, problems can be addressed directly and serves as a basis for therapy adjustment.”). Additional mentions were the availability of sufficient time (n = 3/8.3%), the information gained for the HCP (n = 3/8.3%), the relevance of the findings (n = 2/5.6%) (e.g. “taking findings seriously”, “feeling seen by HCPs”), and the information gain for patients (n = 1/2.8%). Some patients mentioned disadvantages due to the time required (n = 5/13.9%), the confrontation with the condition (n = 3/8.3%), and the in-person presence required (n = 3/8.3%). Individual patients also commented on the need for a report in addition to a personal meeting (n = 1/2.8%), the information gained by HCPs (n = 1/2.8%), and the overly strong influence of the results on therapy (n = 1/2.8%). Furthermore, 4 neutral comments were highlighting that preference depends on the quality of the patient-HCP relationship (n = 4/11.1%).Receiving no report (84 codings 35 patients)The advantages of receiving no report on the results comprised not being confronted with one’s condition (n = 7/20.0% no confrontation with HCPs (n = 2/5.7%), no unnecessary information (n = 1/2.9%), the irrelevance of findings (n = 1/2.9%), and time savings (n = 1/2.9%). The most frequently mentioned disadvantages were lack of access to information (n = 24/68.6%), lack of motivation to complete the questionnaire (n = 9/25.7%) (e.g., ”you may not take the questionnaires as seriously, and you may not see the progress.”), and emotional distress (incl. anxiety, or sadness) caused by not knowing the results (*n* = 4/11.4%).

#### Patient reactions to negative results

##### What do you think a problem (red bar) would trigger in you while at the hospital

Patients were asked what they might experience when they viewed their negative ePROM monitoring result (red bar chart) alone while at the hospital. Negative experiences such as emotional distress (anxiety/sadness) (n = 12/32.4%) (e.g., “This would cause anxiety”) were mentioned most frequently, followed by an indication of a negative disease trajectory (n = 9/24.3%) (e.g., “This would be an indication of a negative outcome”). Also, positive/neutral possible experiences were mentioned as expected reactions to obtaining negative results while at the hospital. Such as a validation of subjective feelings about the disease (n = 8/21.6%) (e.g., “this would confirm my feeling”), no emotional distress (fear/sadness) (n = 4/10.8%), the gain of information for patients (n = 3/8.1%), the possibility of consultation with an HCP if needed (n = 3/8.1%), questioning the correctness of the result (n = 3/8.1%), the reflection on their condition (n = 1/2.7%) (e.g. ”I would try to find an answer to what the problem is.’’), a treatment motivation increase (*n* = 1/2.7%), and the information gained for patients’ health status (*n* = 1/2.7%) was stated.

##### What do you think a problem (red bar) would trigger in you while at home

As possible experiences of receiving negative ePROM results while at home, the majority of patients mentioned a negative impact as, emotional distress (anxiety/sadness) (n = 20/60.6%) (e.g.” The indication of a negative progression would make me sad and anxious. It is alarming. I could not stop thinking about it.”) and the desire for HCP consultation (*n* = 10/30.3%). In addition, patients also expected positive experiences, like the gain of information for patients (*n* = 4/12.1%) (e.g., “Then I know that I need support again”), a treatment motivation increase (*n* = 3/9.1%), professional validation of subjective feelings (*n* = 3/9.1%), and the reflection on their condition (*n* = 1/9.1%), furthermore, several patients would not experience emotional distress (anxiety/sadness) (*n* = 4/12.1%).

#### Support needs

The majority of patients would wish for some sort of support when faced with clinically meaningful results (*n* = 34/94.4%), with most patients (*n* = 33/91.7%) mentioning a desire for HCP consultation (e.g., “I would wish to have a personal conversation about the possible courses of action to change this state.”). Some patients stated that they would not want help at all (*n* = 4/11.1%).

## Discussion

This study evaluated patients’ and HCPs’ understanding of the graphical presentation of ePROM data and found generally high levels of correct interpretation. Overall, our findings indicate that both patients and HCPs were largely able to interpret the graphical ePROM reports correctly, although patients with lower educational levels demonstrated more difficulties. Patients also expressed a high willingness to engage with ePROM assessments, both during hospitalization and at home, suggesting good feasibility of routine monitoring. Consistent with the qualitative insights, patients clearly favored receiving their results through personal interactions with healthcare professionals, emphasizing the value of consultative support, clear communication, and reassurance when clinically meaningful findings arise. While electronic reports were acceptable to a subset of patients, particularly in the home setting, the predominant preference for in-person result delivery underscores the continued importance of interpersonal connection and tailored guidance in the effective use of ePROMs.

A key implication of our findings is the central role of compassion and personal connection in delivering high-quality patient care. Compassion is widely recognized as a core component of care quality and has been linked to improved patient outcomes across clinical contexts. In this light, participants’ strong preference for receiving results in person from their healthcare professionals underscores not only a desire for clarity and trust but also the value they place on personalized and compassionate communication. Face-to-face result delivery ensures that access to support is not revoked at a critical moment, allows for immediate clarification of concerns, and fosters a relational environment in which patients feel understood and cared for. These insights highlight the importance of integrating compassionate, personalized interactions into the delivery of ePROM based clinical information.

Our interpretation accuracy results are quite consistent with the findings of Loth et al., who examined cancer patients’ understanding of graphical presentations of ePROM data. They investigated brain tumor patients’ interpretation accuracy of longitudinal bar charts. Almost all questions on assessing interpretation accuracy were answered correctly by at least 80% of the patients [11]. This is similar to our findings in psychiatric patients, where 80% of patients answered at least 9 out of 12 questions on their results correctly. Slightly different results can be seen in comparison with a previous study of Kujpers et al. [8]. We found higher interpretation accuracy compared to Kujpers et al. [8], who examined cancer patients’ and HCPs’ interpretation accuracy of ePROM results and preferences for graphical presentation styles. Kujipers et al. [12] reported objective understanding rates ranging from 42.8 to 76.7% depending on the type of interpretation (absolute scores, overall change, specific change) and noted that functioning scales were better understood than symptom scales. They also investigated various presentation styles, including coloured and non-coloured bar and line charts and a heat map. While interpretation accuracy did not significantly vary across these styles, patients who viewed colored charts demonstrated a preference for bar charts. The authors suggest that their lower objective interpretation accuracy compared to the literature is due to the different types of graphical presentation styles, which could also be the case in comparison to our study [12]. Similar outcomes are shown in a study by Brundage et al. [24], which investigated cancer patients’ interpretation of health-related Quality of Life data in multiple formats (e.g. textual, line graphs, change bars). Patients preferred a simple linear representation of group mean health-related Quality of Life scores and reported a lower rate of correct responses than our study, but a smaller range of correct answers (85–98% vs. 25–100%) [24], which could be due to different presentation styles. Recent systematic reviews highlight that while bar charts and line graphs are commonly used for PROM visualization, no single format achieves uniformly superior interpretation accuracy. Enhancements such as color coding, clear scale direction, and descriptive labels significantly improve usability [9].

Our study establishes significant correlations between the objective understanding of graphical ePROM presentation and educational level, mirroring findings from previous studies involving oncological patients [12, 24]. This association can be understood in terms of numeracy and graph literacy, as individuals with lower educational attainment may face greater difficulty interpreting numerical and graphical information [25].

Additionally, patients in this study showed greater willingness to complete the questionnaires in the hospital setting than at home, contrasting with trends observed in cancer patients [12], possibly due to the poorer health status of oncology patients during an inpatient stay. Implementation studies using ePROM dashboards in real-world clinical settings demonstrate that when clinicians engage with PROM visualizations, can futher improve patients’ adherence to completing PROMs, supporting the feasibility of regular monitroing [26]. HCPs in our study demonstrated similar interpretation accuracy compared to findings from Kuijpers et al. who reported 78% interpretation accuracy for medical specialists and 74% for nurses and other health professionals. In our study, 85% of HCPs answered no more than one question incorrectly. Kuijpers et al. also reported a wider range of interpretation accuracy, with 52.9 to 94.1%, while our results ranged from 68.8% to 100% [12]. Brundage et al. [27] reported better interpretation accuracy for HCPs (90–100%) than in our study. This difference may be due to the types of HCPs included. The distribution of HCPs roles largely reflect the composition of the actual treatment team, with a slight overrepresentation of nursing staff. This aligns with their proportionally larger presence in daily clinical work and patient contact. While the study of Brundage et al. [27] focuses only on oncologists, our sample included various HCP professions.

Furthermore, our qualitative analysis can provide more insights into patients’ preferred report methods and their causes. Patients seem to find several advantages to paper reports and personal meetings, while they find barely any advantages if receiving no report. Paper reports were valued for their physical availability and ease of access, yet concerns included risks of loss of paper reports and a lack of personal contact. Electronic reports appealed for their quick access and good availability but presented challenges for those less technologically adept, and concerns with data protection were noted. Despite a lack of privacy and time constraints, report during ward rounds was valued for fostering interpersonal connection and information exchange between HCPs. Personal meetings were evaluated most positively, with the frequently mentioned positive effects through the consultation itself, the good comprehensibility of ePROM data, and privacy. Only a few patients mentioned disadvantages due to the time required. These findings align with Kuijpers et al. [12], who reported that nearly half of the patients in their study (49.7%) preferred oral report from an HCP on ePROM results. In addition, Carlier et al. [6] investigated the provision of routine reported monitoring and noted that discussing ePROM outcomes positively impacts communication between patients and HCPs. One plausible explanation for patients’ preference for personal meetings relates to the reduction of cognitive load when processing emotionally meaningful health information. In-person consultations provide emotional safety, foster trust, and offer immediate opportunities for clarification, improving understanding. Moreover, may empathic communication can alleviate anxiety related to potentially negative results, further strengthening the preference for face-to-face interaction. Taken together, these cognitive, educational, and emotional factors help explain why patients report a strong desire for personal, face-to-face feedback. It not only supports a clearer understanding but also enables immediate clarification of questions and provides empathic relief, ultimately enhancing the overall quality and personalization of care.

The results of our study indicate patients are willing to engage with ePROMs and prefer receiving their ePROM report through a personal meeting with an additional paper report, while an electronic report serves as a valuable alternative for at-home report. Patients also expressed a desire for support when receiving negative results, underscoring the need for accessible assistance options. Synthesized evidence further suggests that feedback of PROM data can drive better self-management, shared decision-making, and personalized care planning [28, 29]. These findings reinforce the clinical relevance of our results, showing that patients value personal, clear, and supportive delivery of ePROM results. Furthermore, a study of Greenhalgh et al. indicates that PROMs can stimulate patients’ self-reflection on their symptoms, bringing relevant topics into consultations with healthcare professionals. Clinicians, in turn, are alerted to issues highlighted by the PROM data, enabling more targeted and effective patient care [28].

One limitation of our study is the use of a single graphical format (bar charts) for the presentation of results. Although bar charts were selected based on prior evidence [16], we did not explore different variations of bar chart designs that may have provided further insights into optimal design choices. Our findings, therefore, offer an initial foundation that should be expanded in subsequent research. Additionally, the small sample size, as the single-center design, limited the statistical robustness of our quantitative results. It is also important to note that the educational level of our sample roughly reflects the distribution in the Austrian general population [30], but there is likely a slight underrepresentation of individuals with lower educational attainment. However, a key strength of our study was that participating patients had prior experience with ePROMs, enhancing their understanding of the study’s purpose and offering a more accurate view of the patients’ perspective.

In addition to these strengths and limitations, our findings provide important implications for the future development of ePROM feedback. The results indicate that returning ePROM information directly to patients may increase their engagement and encourage a more active role in their treatment. Building on this, future research should examine whether different graphical formats or more personalized feedback strategies could further boost patients’ adherence to ongoing symptom monitoring. Additionally, it will be crucial to explore in greater depth how patients interpret information about potential benefits or risks when receiving ePROM feedback, and how these insights may influence their motivation for therapy. These aspects may differ across diagnostic groups or levels of illness severity, highlighting the need for tailored approaches to maximize the clinical utility of ePROM reporting. In practice, this means that clinicians could routinely review ePROM results with patients during ward rounds or follow-up visits, while system designers ensure that visualization tools are intuitive, consistent, and tailored to the needs of psychosomatic patients. This study demonstrates that implementing ePROM in psychosomatic medicine is well-received by patients. They are willing to complete the questionnaires and show interest in their results. A practical recommendation is to clarify the scale direction to improve the accurate interpretation of graphical ePROM data. Moreover, their preferred method of receiving reports is through personal meetings. This approach could be integrated into daily clinical routines in psychosomatic wards. Overall, patients already understand the presented information quite well; however, clearer scale markers and structured feedback conversations can further aid healthcare professionals and system designers in maintaining this high level of clarity.

## Conclusion

This study investigated patients’ and HCPs’ understanding of the graphical presentations of ePROM results, as well as patients’ perspectives and preferences for report methods. Our findings suggest that both patients and HCPs generally demonstrate high accuracy in interpreting ePROM data presented as bar charts within a psychosomatic setting. Additionally, most patients expressed a desire for access to their results, with the preference for receiving there report directly from HCPs along with support in cases of negative report. Based on these qualitative findings, future studies could quantitatively assess the impact of delivering ePROM results to patients through a personal meeting supplemented by an additional report as a report method.

## Supplementary Information

Below is the link to the electronic supplementary material.

Supplementary Material 1

## Data Availability

The data that support the findings of this study are available on reasonable request from the corresponding author. The data are not publicly available due to privacy or ethical restrictions.

## Glossary

Healthcare professionals (HCPs): Professionals working at the psychosomatic ward (nurses, psychologists, psychotherapists, medical doctor, social worker, physiotherapist).
Patient-reported outcomes (PROs): Measure to evaluate the impact of health conditions and their treatment from the patient’s perspective.
Electronic PRO monitoring (ePROM): Involves collecting, analyzing, and documenting patient self-ratings, enabling treatment monitoring, screening, quality assurance, and research.

## References

[1] Gault RH (1907) A History of the Questionnaire Method of Research in Psychology. Pedagogical Seminary 14(3):366–383

[2] Boswell JF, Kraus DR, Miller SD, Lambert MJ (2015) Implementing routine outcome monitoring in clinical practice: benefits, challenges, and solutions. Psychother Res 25(1):6–19 23885809 10.1080/10503307.2013.817696

[3] Jensen RE, Snyder CF, Abernethy AP, Basch E, Potosky AL, Roberts AC et al (2014) Review of electronic patient-reported outcomes systems used in cancer clinical care. J Oncol Pract 10(4):e215–e222 24301843 10.1200/JOP.2013.001067 PMC4094646

[4] Meirte J, Hellemans N, Anthonissen M, Denteneer L, Maertens K, Moortgat P, Van Daele U (2020) Benefits and Disadvantages of Electronic Patient-reported Outcome Measures: Systematic Review. JMIR Perioper Med 3(1):e15588 33393920 10.2196/15588 PMC7709853

[5] Gilbody SM, House AO, Sheldon T (2002) Routine administration of Health Related Quality of Life (HRQoL) and needs assessment instruments to improve psychological outcome–a systematic review. Psychol Med 32(8):1345–1356 12455933 10.1017/s0033291702006001

[6] Carlier IV, Meuldijk D, Van Vliet IM, Van Fenema E, Van der Wee NJ, Zitman FG (2012) Routine outcome monitoring and feedback on physical or mental health status: evidence and theory. J Eval Clin Pract 18(1):104–110 20846319 10.1111/j.1365-2753.2010.01543.x

[7] Metz MJ, Franx GC, Veerbeek MA, de Beurs E, van der Feltz-Cornelis CM, Beekman ATF (2015) Shared Decision Making in mental health care using Routine Outcome Monitoring as a source of information: a cluster randomised controlled trial. BMC Psychiatry 15(1):313 26666295 10.1186/s12888-015-0696-2 PMC4678650

[8] Campbell N, Ali F, Finlay AY, Salek SS (2015) Equivalence of electronic and paper-based patient-reported outcome measures. Qual Life Res 24(8):1949–1961 25702266 10.1007/s11136-015-0937-3

[9] Albers EAC, Fraterman I, Walraven I, Wilthagen E, Schagen SB, van der Ploeg IM et al (2022) Visualization formats of patient-reported outcome measures in clinical practice: a systematic review about preferences and interpretation accuracy. J Patient Rep Outcomes 6(1):18 35239055 10.1186/s41687-022-00424-3 PMC8894516

[10] Snyder C, Smith K, Holzner B, Rivera YM, Bantug E, Brundage M (2019) Making a picture worth a thousand numbers: recommendations for graphically displaying patient-reported outcomes data. Qual Life Res 28(2):345–356 30306533 10.1007/s11136-018-2020-3 PMC6363861

[11] Loth FL, Holzner B, Sztankay M, Bliem HR, Raoufi S, Rumpold G, Giesinger JM (2016) Cancer patients’ understanding of longitudinal EORTC QLQ-C30 scores presented as bar charts. Patient Educ Couns 99(12):2012–2017 27506581 10.1016/j.pec.2016.08.004

[12] Kuijpers W, Giesinger JM, Zabernigg A, Young T, Friend E, Tomaszewska IM et al (2016) Patients’ and health professionals’ understanding of and preferences for graphical presentation styles for individual-level EORTC QLQ-C30 scores. Qual Life Res 25(3):595–604 26353905 10.1007/s11136-015-1107-3 PMC4759250

[13] Snyder CF, Smith KC, Bantug ET, Tolbert EE, Blackford AL, Brundage MD (2017) What do these scores mean? Presenting patient-reported outcomes data to patients and clinicians to improve interpretability. Cancer 123(10):1848–1859 28085201 10.1002/cncr.30530 PMC5419857

[14] Oerlemans S, Arts LP, Horevoorts NJ, van de Poll-Franse LV, Am (2017) I normal? The Wishes of Patients With Lymphoma to Compare Their Patient-Reported Outcomes With Those of Their Peers. J Med Internet Res 19(8):e288 28811271 10.2196/jmir.7079 PMC5575418

[15] Roca M, Vives M, López-Navarro E, García-Campayo J, Gili M (2015) Cognitive impairments and depression: a critical review. Actas Esp Psiquiatr 43(5):187–193 26320897

[16] Kristensen S, Mainz J, Baandrup L, Bonde M, Videbech P, Holmskov J, Bech P (2018) Conceptualizing patient-reported outcome measures for use within two Danish psychiatric clinical registries: description of an iterative co-creation process between patients and healthcare professionals. Nord J Psychiatry 72(6):409–419 30015541 10.1080/08039488.2018.1492017

[17] Tolbert E, Brundage M, Bantug E, Blackford AL, Smith K, Snyder C, Board PRODPSA (2019) proportion: approaches for displaying patient-reported outcome research study results as percentages responding to treatment. Qual Life Res 28(3):609–620 30498892 10.1007/s11136-018-2065-3 PMC6387635

[18] Damman OC, Verbiest MEA, Vonk SI, Berendse HW, Bloem BR, de Bruijne MC, Faber MJ (2019) Using PROMs during routine medical consultations: The perspectives of people with Parkinson’s disease and their health professionals. Health Expect 22(5):939–951 31199574 10.1111/hex.12899 PMC6803413

[19] Geerards D, Pusic A, Hoogbergen M, van der Hulst R, Sidey-Gibbons C (2019) Computerized Quality of Life Assessment: A Randomized Experiment to Determine the Impact of Individualized Feedback on Assessment Experience. J Med Internet Res 21(7):e12212 31298217 10.2196/12212 PMC6657452

[20] Egeter J, Hüfner K, Sztankay M, Holzner B, Sperner-Unterweger B (2018) Implementation of an electronic routine outcome monitoring at an inpatient unit for psychosomatic medicine. J Psychosom Res 105:64–71 29332636 10.1016/j.jpsychores.2017.12.009

[21] Holzner B, Giesinger JM, Pinggera J, Zugal S, Schöpf F, Oberguggenberger AS et al (2012) The Computer-based Health Evaluation Software (CHES): a software for electronic patient-reported outcome monitoring. BMC Med Inf Decis Mak 12:126 10.1186/1472-6947-12-126 PMC3529695 23140270

[22] Broadbent E, Petrie KJ, Main J, Weinman J (2006) The brief illness perception questionnaire. J Psychosom Res 60(6):631–637 16731240 10.1016/j.jpsychores.2005.10.020

[23] Corp (2020) I. IBM SPSS Statistics for Macintosh, Version 28.0. NY: IBM Corp, Armonk. [

[24] Brundage M, Feldman-Stewart D, Leis A, Bezjak A, Degner L, Velji K et al (2005) Communicating quality of life information to cancer patients: a study of six presentation formats. J Clin Oncol 23(28):6949–6956 16192583 10.1200/JCO.2005.12.514

[25] Durand MA, Yen RW, O’Malley J, Elwyn G, Mancini J (2020) Graph literacy matters: Examining the association between graph literacy, health literacy, and numeracy in a Medicaid eligible population. PLoS ONE 15(11):e0241844 33175891 10.1371/journal.pone.0241844 PMC7657552

[26] Pasma A, van Lint C, Hollander MA, Bruinsma SM, Peters IA (2024) Healthcare providers’ use of dashboards with patient reported outcomes reinforces patients to fill out patient reported outcome measures. Digit Health 10:20552076241293975 40290730 10.1177/20552076241293975 PMC12032432

[27] Brundage MD, Smith KC, Little EA, Bantug ET, Snyder CF (2015) Communicating patient-reported outcome scores using graphic formats: results from a mixed-methods evaluation. Qual Life Res 24(10):2457–2472 26012839 10.1007/s11136-015-0974-y PMC4891942

[28] Greenhalgh J, Dalkin S, Gooding K, Gibbons E, Wright J, Meads D, Health Services and Delivery Research (2017). Functionality and feedback: a realist synthesis of the collation, interpretation and utilisation of patient-reported outcome measures data to improve patient care. Southampton (UK): NIHR Journals Library Copyright © Queen’s Printer and Controller of HMSO 2017. This work was produced by Greenhalgh. under the terms of a commissioning contract issued by the Secretary of State for Health. This issue may be freely reproduced for the purposes of private research and study and extracts (or indeed, the full report) may be included in professional journals provided that suitable acknowledgement is made and the reproduction is not associated with any form of advertising. Applications for commercial reproduction should be addressed to: NIHR Journals Library, National Institute for Health Research, Evaluation, Trials and Studies Coordinating Centre, Alpha House, University of Southampton Science Park, Southampton SO16 7NS, UK 28121094

[29] Wittich L, Tsatsaronis C, Kuklinski D, Schöner L, Steinbeck V, Busse R, Rombey T (2024) Patient-Reported Outcome Measures as an Intervention: A Comprehensive Overview of Systematic Reviews on the Effects of Feedback. Value Health 27(10):1436–1453 38843978 10.1016/j.jval.2024.05.013

[30] Statistik Austria. Bildungsstand der Bevölkerung (2024) Available from: https://www.statistik.at/statistiken/bevoelkerung-und-soziales/bildung/bildungsstand-der-bevoelkerung.

