# Supplementary material for: A mixed methods study on patients’ and healthcare professionals’ understanding of the graphical presentation of patient-reported outcome data at an inpatient unit for psychosomatic medicine
Source: J Patient Rep Outcomes. 2026 Apr 11;10:82. doi: 10.1186/s41687-026-01061-w (PMC13194904; doi:10.1186/s41687-026-01061-w)
Supplement: Supplementary file 1 — Supplementary Material 1 [file 41687_2026_1061_MOESM1_ESM.docx]

### Declarations

### Funding

No funding was received for conducting this study.

### Ethics approval and consent to participate

Ethical approval of the study was obtained from the institutional ethics committee [AN-2014-0012].

### Competing interest

Bernhard Holzner is the owner of the intellectual property rights of the CHES software. The other authors declare no conflicts of interest.

### Acknowledgment

All clinical personnel at the ward for psychosomatic Medicine for their continuous support

### Author Contributions

Hüfner K, Egeter J, Loth FLC, Holzner B, and Sperner-Unterweger B contributed to the conception or design of the work. Thurner AMM, Gross F, and Schurr T contributed to data acquisition and analysis. Thurner AMM and Giesinger JM interpreted the data for the work. Thurner AMM and Gross F drafted the work, and Hüfner K, Egeter J, Loth FLC, Holzner B, Sperner-Unterweger B, and Giesinger JM reviewed it critically for important intellectual content. All authors approved the final version to be published and agreed to be accountable for all aspects of the work in ensuring that questions related to the accuracy or integrity of any part of the work are appropriately investigated and resolved.

### Availability of data and material

The data that support the findings of this study are available on reasonable request from the corresponding author. The data are not publicly available due to privacy or ethical restrictions.
